# Impediments to compliance during filariasis mass drug administration—Observations and recommendations to accelerate filariasis elimination in India

**DOI:** 10.3389/fpubh.2024.1384131

**Published:** 2024-06-17

**Authors:** Philip Raj Abraham, Vijesh Sreedhar Kuttiatt, Manju Rahi, Ashwani Kumar

**Affiliations:** ^1^Unit of Molecular Epidemiology, Indian Council of Medical Research (ICMR)-Vector Control Research Centre, Puducherry, India; ^2^Unit of Clinical and Molecular Medicine, Indian Council of Medical Research (ICMR)-Vector Control Research Centre, Puducherry, India; ^3^Indian Council of Medical Research (ICMR)-Vector Control Research Centre, Puducherry, India; ^4^Saveetha University, Chennai, India

**Keywords:** lymphatic filariasis, outliers, MDA, IDA, ineligible, compliance

## Introduction

Lymphatic filariasis (LF), with its crippling manifestations of lymphedema, causes considerable morbidity and chronic suffering. The new, ambitious global target for the elimination of LF as a public health problem is 2030. It was prevalent in 81 countries at the beginning of the Global Programme to Eliminate Lymphatic Filariasis (GPELF) in 2000 and currently, 72 countries are endemic with 120 million people at risk. The World Health Organization (WHO) has recommended mass drug administration (MDA) for the interruption of LF transmission in endemic areas. A total of five to six rounds of MDA with >65% treatment coverage are necessary for achieving the elimination threshold (< 1% microfilaraemia or < 2% antigenaemia). Hypothetically, achieving the LF elimination in five to six MDA rounds with 65% treatment coverage is challenging mainly due to suboptimal drug compliance (1). Overestimation of drug compliance is suspected in the absence of objective criteria to verify actual drug consumption, even though supervised treatment is recommended. India, which is highly endemic for LF with 333 endemic districts in 20 states/union territories, will be continuing MDA in 176 districts (2). Realizing that MDA will remain the primary intervention for LF elimination, we examined the reasons for sub-optimal compliance during the MDA rounds and various supplementary measures available as supportive interventions for transmission reduction, which would enable the achievement of LF elimination targets in India.

As investigators of a large triple drug (Ivermectin, DEC, and Albendazole) MDA trial in southern India, we realized that a significant proportion of individuals remain untreated (the outlier population) following MDA and the subsequent mop-up rounds due to various reasons. These “outliers” include (1) eligible individuals who intentionally do not consume anti-filarial drugs despite a number of treatment rounds, i.e., systematic non-compliance (3), and those who miss treatment unintentionally due to their mere absence or as temporary migrants at the time of MDA; (2) the “ineligibles” who cannot be treated safely, e.g., pregnant women, children < 2 years of age, and those suffering from chronic diseases; and (3) the older adult population (60 years and above; Ministry of Statistics and Programme Implementation (MOSPI), Government of India). All of them provide a wide window of opportunity to improve drug compliance. Accessing and treating the outliers in our opinion can greatly ramp up the effectiveness of MDA. This article highlights our observations and further recommendations to accelerate filariasis elimination in India.

## The eligible population of MDA

Systematic non-compliance among the eligible population will pose a health risk to the entire community. Moreover, they carry higher filarial infection rates than drug-compliant populations (4, 5). Although the causes of systematic non-compliance among men and women are different (6), with effective methods of communication and social mobilization, this category of the eligible population can become drug compliant during MDA. In our own experience and that of others, community non-compliance partly stems from the fear of adverse events, ignorance, and misconceptions about the drugs (7–9). In addition, there is “community fatigue”, as people perceive a lack of benefit from a prolonged and repeated intervention. Strategies such as supervised treatments, effective adverse events management, health education, community mobilization, and engagement of religious leaders have resulted in an attitude change and improvement of drug compliance during the triple drug study in Yadgir district, India, which had a high prevalence of antigaenemia (25.6%) and microfilaraemia (6.7%), despite 14 rounds of MDA (8, 9). The findings of this study were instrumental in the introduction of triple-drug MDA to accelerate LF elimination in India (2, 10). These strategies could be suitably adopted in other similar settings. However, covering the migrants under the MDA programmes is a challenging task. A study of migrant labor settlement in Kerala showed that 3.6% of the migrants tested positive for filarial antigen (11). Migrant workers from an LF endemic area showed an overall filarial antigenaemia prevalence of 18.3% in Kuwait (12). Correspondingly, the reasons for not receiving the treatment (37.1%) were either being absent or traveling during MDA (13). It is pertinent to mention that post-MDA assessments showed reason for non-coverage was due to missing these people (26.6–50.3%) by the drug administrator (14). Furthermore, 4.7–33.6% of individuals were non-compliant to drugs due to their absence at the time of drug distribution, as the drugs were received by family members. The treatment gap due to missed opportunities and temporary migration could be addressed by rescheduling the MDA, adopting effective mopping-up strategies, and line listing, coupled with arrangements for the treatment of migrants upon their return or in the present place of living through local health services.

## The ineligible population of MDA

At any given time, the “ineligibles” for MDA would constitute 10–20% of the population (2). In an endemic area, depending upon the microfilaraemia prevalence rate, it is reasonable to assume that some of these individuals would be infected. Apart from their role in furthering transmission, it is also unethical to leave such infected individuals in the community untreated. For temporarily ineligible persons, guidelines are lacking for their treatment following an MDA round. For example, a pregnant woman with microfilariae in the blood serves as a persistent source of infection in the community and must wait until a subsequent round of MDA for drug consumption. In addition, filarial infection during pregnancy has serious implications on the child's immune response and consequently its susceptibility to filarial infection (15, 16). Therefore, it is important to screen for microfilariae during pregnancy and conduct a line listing of such microfilaraemic pregnant women. Their treatment postpartum is highly desirable as the early treatment reverses subclinical lymphatic damage in children born to microfilaraemic mothers (17, 18). Since LF is acquired during childhood, children aged 5 years and above are also treated with DEC and Albendazole during MDA. This is because one-third of children acquire the infection before they reach the age of 5 years (19). Filarial antigen showed an increased prevalence among 2- to 4-year-old children (6% to more than 30%, respectively) (20). In addition, seroepidemiology has demonstrated an increasing prevalence of filarial antibodies in a population of children as young as 6 months of age (21). Since children < 2 years are not included in MDA, all children aged between 18 and 24 months may be tested and treated upon attaining 2 years of age with DEC and Albendazole combination (note: ivermectin is contraindicated in children under 5 years of age and under 15 kg body weight).

Chronic diseases are the leading causes of disability and premature death, especially in older adults. Approximately 21% of the Indian older adult population reportedly have at least one chronic disease (22). For those with chronic diseases, testing and treating with antifilarials under medical supervision would be appropriate. As a supportive intervention, ineligibles may be provided with long-lasting insecticide-treated nets (LLINs). Additionally, the misclassification of “eligible” as “ineligibles” by drug distributors could also lead to a significant number of people remaining out of the ambit of MDA (14). Therefore, standard guidelines for eligibility criteria and training of the drug distributors are necessary.

## The older adult population

According to the Ministry of Statistics and Programme Implementation (MOSPI), Government of India, individuals aged 60 years and above are considered as older adult population. They are vulnerable to chronic diseases, and treatment-seeking is relatively low among them in poor households (22, 23). Furthermore, the prevalence of communicable diseases among older adults will be high (24.9%) (24). During MDA, a majority of the older adult population refuse the drugs due to fear of adverse events, the need to consume a greater number of drugs, and the belief that they should not take drugs when they are not ill. Moreover, the drug distributor also ignores them as it is very difficult to convince this segment of the population. Furthermore, elders with chronic diseases are also dropped from the MDA. The number of older adult population accumulates in each round, and the infected among them will become a significant source of infection as they are left out in every round of MDA. Therefore, it is important to test these outliers, and the infected among them may be provided with LLINs, similar to the ineligible population.

## Conclusion

As it is a well-known fact that the entire community cannot be covered during MDA, there is an urgent need to devise strategies to cover outlier populations. In this context, effective IEC will play a major role in covering eligible populations before MDA programmes. This is already known and it was successfully utilized in most countries. Regarding ineligible populations such as those below the age of eligibility and pregnant women, compliance issues are not voluntary by an individual, as it is a temporary condition. Generally, children and pregnant mothers are under the observation of ‘Anganwadi workers' (AWW) in India. The AWW may cover this population as and when they become eligible. However, individuals with chronic disease and the older adult are at risk and hence they may be tested for filarial infection if any. In conclusion, it is clear that MDA as a standalone and “one size fits all” strategy has limitations in certain settings (25). Therefore, targeted strategies to access, test, and treat the outliers, especially in highly endemic areas are necessary. As a successful example, testing and treating, vector control, DEC medicated salt, and active community participation were crucial in LF elimination from Japan (e.g., Japanese “Araragama spirit”), South Korea, and China (26, 27). The national programme may consider the implementation of strategies such as microplanning for vector control, environmental engineering, effective coverage of outliers, and DEC-fortified salt distribution in areas with persistent transmission to achieve global LF elimination in India.

## Author contributions

PA: Conceptualization, Writing – original draft, Writing – review & editing. VK: Conceptualization, Writing – original draft, Writing – review & editing. MR: Writing – review & editing. AK: Conceptualization, Writing – review & editing.
